# Protein Corona Prevents TiO_2_ Phototoxicity

**DOI:** 10.1371/journal.pone.0129577

**Published:** 2015-06-17

**Authors:** Maja Garvas, Anze Testen, Polona Umek, Alexandre Gloter, Tilen Koklic, Janez Strancar

**Affiliations:** 1 Jožef Stefan Institute, Ljubljana, Slovenia; 2 Jožef Stefan International Postgraduate School, Ljubljana, Slovenia; 3 NAMASTE Center of Excellence, Ljubljana, Slovenia; 4 Laboratoire de Physique des Solides, Université Paris Sud, CNRS UMR 8502, F-91405, Orsay, France; Argonne National Laboratory, UNITED STATES

## Abstract

**Background & Aim:**

TiO_2_ nanoparticles have generally low toxicity in the *in vitro* systems although some toxicity is expected to originate in the TiO_2_-associated photo-generated radical production, which can however be modulated by the radical trapping ability of the serum proteins. To explore the role of serum proteins in the phototoxicity of the TiO_2_ nanoparticles we measure viability of the exposed cells depending on the nanoparticle and serum protein concentrations.

**Methods & Results:**

Fluorescence and spin trapping EPR spectroscopy reveal that the ratio between the nanoparticle and protein concentrations determines the amount of the nanoparticles’ surface which is not covered by the serum proteins and is proportional to the amount of photo-induced radicals. Phototoxicity thus becomes substantial only at the protein concentration being too low to completely coat the nanotubes’ surface.

**Conclusion:**

These results imply that TiO_2_ nanoparticles should be applied with ligands such as proteins when phototoxic effects are not desired - for example in cosmetics industry. On the other hand, the nanoparticles should be used in serum free medium or any other ligand free medium, when phototoxic effects are desired – as for efficient photodynamic cancer therapy.

## Introduction

Titanium dioxide (TiO_2_) became an indispensable chemical, widely used as a white pigment or a UV light scavenger in plastic and paper industry, in cosmetics, in food industry as well as in pharmaceutics. The application of the TiO_2_ material relies on the two of its properties: absorption of the UV radiation and photocatalysis. The latter is triggered by the absorption of the UV light that induces reactive oxygen species formation that can damage biological structures. Nano forms of TiO_2_ with large ratio between surface area and volume amplify the aforementioned properties of the bulk TiO_2_ [1] making these formulations an excellent self-cleaning and self-sterilizing materials as well as efficient anticancer therapeutics [2,3]. With respect to the large number of applications of the TiO_2_ nanostructures the investigation of the corresponding toxicity and phototoxicity is essential for our safety.

TiO_2_ nanoparticles have generally low toxicity, with LC50 greater than 100 μg/mL when applied in a dispersion together with serum proteins and measured in *in vitro* cell culture systems [4,5] On the other hand, when irradiated by UV light, TiO_2_ generates radicals that certainly do affect biological systems. However, since the same particles accumulate in tissues like the testicle [6], the question about their phototoxicity becomes very relevant [7]. Nevertheless, TiO_2_ formulations that include substantial amount of nano forms are used in sunscreens and food additives although declared as a safe and natural material. With the tendency of using ever smaller forms of TiO_2_ nanoparticles contributing to a better transparency, the amount of nano forms is increased further and so the phototoxicity [8,9]. On the other hand, highly phototoxic TiO_2_ nanoparticles could be used beneficially. They could improve photodynamic cancer therapy, which hasn’t been brought into human therapy yet [10]. Photoactivated TiO_2_ nanoparticles, with diameters of around 100 nm, already showed promising results in breast cancer epithelial cells, but very large concentrations had to be applied [11]. Better understanding of the mechanism of TiO_2_ nanoparticles phototoxicity can therefore lead to more efficient and safer applications of these nanomaterials.

The mechanism of the phototoxic effect of TiO_2_ nanoparticles has been investigated by a multi-parametric analysis recently [8]. The authors concluded that the phototoxicity of TiO_2_ nanoparticles originates mainly in the reactive oxygen species (ROS) formation. The latter is found to depend on the size of the nanoparticles as well as on the biomolecule absorption. Namely, hydroxyl radicals produced on the surface of the nanoparticles can damage biomolecules such as DNA or proteins more likely if these biomolecules are bound to the surface of nanoparticles. On the contrary, if these molecules are further away from the nanoparticle surface the radicals are more likely to be scavenged by the various antioxidants thus being less harmful to the cell content. Although albumin, the most abundant serum protein, has been recently recognized as a radical scavenger, responsible for protection of other essential proteins against oxidation [12], its role in the phototoxicity of TiO_2_ nanotubes has not been addressed yet.

Here we therefore focus on the phototoxic effect of the TiO_2_ nanotubes (TiO_2_NTs) and show that the phototoxicity becomes substantial at the concentrations of proteins when the nanotubes are not fully occupied by the proteins. Namely, the phototoxic effect is found to be proportional to the surface of TiO_2_NTs, not covered by proteins. By spin trapping EPR spectroscopy we confirm that proteins are able to bind to the nanotube surfaces and efficiently trap photoinduced hydroxyl radicals. These results have two important implications. When phototoxic effects are not desired, for example in case of sunscreens, TiO_2_ nanoparticles should be applied with ligands such as proteins or coated with polymers, whose oxidation results in a non-toxic product. On the other hand, when strong radical generation is desired like in an efficient photodynamic cancer therapy, the nanoparticles should be used in a serum free medium or any other nanoparticle ligand free medium.

## Materials and Methods

### Synthesis of the TiO_2_ nanotubes (TiO_2_-NTs)

Sodium titanate nanotubes (NaTiNTs) were first synthesized as reported recently [13]. Next, sodium ions were removed by an ion-exchange process by dispersing 2.5 g of NaTiNTs into 200 mL of 0.1 M HCl(aq) [14]. The prepared dispersion was stirred at room temperature for 1 h, centrifuged to separate the solid material from the solution, which was removed. The dispersing, centrifuging and separating steps were repeated for two more times. At the end, the solid material was washed with 200 mL of distilled water, 100 mL of EtOH and dried overnight at 100°C. Finally, the isolated hydrogen titanate nanotubes were transformed into TiO_2_ nanotubes (TiO_2_-NTs) by thermal treatment in air at 375°C for 10 h.

### Characterization of TiO_2_-NTs

The morphology and dimensions of the prepared TiO_2_-NTs were investigated with a transmission electron microscope (TEM, Jeol 2100, 200 keV). The XRD was performed on a Bruker AXS D4 Endeavor diffractometer using CuKα1 with a wavelength λ = 1.5406 Å. The STEM-HAADF (high angle annular dark field) and STEM-EELS measurements were performed using a C3/C5 spherical aberration-corrected microscope Nikon U-STEM working at 100 keV.

### In vitro cell viability/ cytotoxicity studies/ phototoxicity studies

TiO_2_-NTs powder was diluted in 1M potassium hydroxide and sonicated with the sonicator (MISO XVIX Ultrasound liquid Processors) for 15 min, with a 10-seconds long pulse ON and a 5-seconds long OFF with an amplitude of 90 on ice bath (power 20–30 W). To ensure sterility we sterilize the nanomaterial dispersion in an autoclave at 121°C for 30 min (Kambič Parni sterilizator A-21 CAV) and cool it to room temperature overnight.

### Cell viability test

To determine potentially reduced cell viability due to the toxicity of the TiO_2_NTs calorimetric cell viability MTS assay (CellTiter96 AQueous One Solution Cell Proliferation Assay, Promega, Madison, WI) was used. The solution reagent contains tetrazolium compound MTS [3-(4,5-dimethylthiazol-2-yl)-5-(3-carboxymethoxyphenyl)-2-(4-sulfophenyl)-2H-tetrazolium] and an electron coupling reagent (phenazine ethosulfate; PES). The MTS tetrazolium compound is reduced by living cells into a colored formazan product that is soluble in tissue culture medium. The method is based on the accumulation of the colored formazane products inside living cells by measuring optical density of a sample. Nanoparticle dispersions were freshly prepared and diluted with the cell culture medium (DMEM, Gibco—Life tehnologies) with 10% of the fetal bovine serum (FBS, PPA Laboratories GmbH) to the desired concentrations. Approximately 5000 MCF-7 cells/100 μL were seeded in four 96-well culture plates in parallel (CellGrade premium, BRAND) in DMEM medium with FBS and antibiotics (Sigma Aldrich, St. Louis) at 37°C and 5% of CO_2_. After an overnight incubation (attachment phase) the cells were washed with fresh phosphate-buffered saline (PBS, Gibco—Life tehnologies) to remove dead cells and the fresh medium with the corresponding amount of well dispersed TiO_2_-NTs was added to the cells. Cells were exposed to a dispersion of nanotubes with mass concentration of 1, 5, 50, 100, 500 or 1000 μg/mL and incubated in dark overnight. The control experiments were performed without nanoparticles as well as with nanoparticles in medium but without cells. During the second day, two plates were irradiated with UV light (UV LED lamp with 1.6 W/m^2^, wavelength of 365 nm, 15 min irradiation every 3 h for 24 h, resulting in 2 h of total irradiation), other two plates were incubated in dark. During the third day 20 μL of the MTS reagent was added to each culture plate. After 2 h of incubation, the absorbance at 490 nm was measured on microplate reader Infinite M1000 (Tecan, Männendorf, Switzerland) at room temperature, using top mode. Absorbance, which measures the quantity of formazan product, is proportional to the number of viable cells in the cell culture. The results were presented as the absorbance ratio of treated to control cells. Control experiment and irradiated samples without nanoparticles were repeated in approximately 130 different wells, samples with nanoparticle treatment were repeated in approximately 80 different wells. All measurements were performed within 7 different passages of cells. All the results are presented in a form of a boxplot, where the solid black line in a box marks the median value of the MTS test, while the red line marks the mean. The box indicates the range between 25^th^ and 75^th^ percentile. Whiskers (error bars) above and below the box indicate the range between 10^th^ and 90^th^ percentiles. The outliers are shown as closed black circles. Since normality test (Shapiro-Wilk) failed, Mann-Whitney U statistics was employed instead of t-test.

### Photocatalytic activity of the TiO_2_-NTs

The photocatalytic activity of the synthesized TiO_2_-NTs were compared with the photocatalytic activity of the frequently investigated nanoparticles (P25 from Degussa) as a function of UV wavelength (UV led lamps). The photocatalytic activity was determined via the numbers of the hydroxyl radical, trapped with a spin trap 5-(Diethoxyphosphoryl)-5-methyl-1-pyrroline-N-oxide (DEPMPO, Alexis, Lausen) used as purchased without further purification and stored at -80°C. To exclude possible (false positive) artefacts due to decomposition of a spin trap or due non-radical reactions, which could give the same spectra as OH radical adducts, the same measurements were repeated also in the presence of 30% (v/v) of ethanol (EtOH, Merck AG, Germany). Namely, ethanol captures the OH radical and transforms it to the ethyl radical, which captured proved the primary generation of the hydroxyl radicals [15]. The stock suspension of TiO_2_-NTs or P25 in 1M KOH (pH 11) was sonicated for 15 min (10 s pulse-ON time, 10 s pulse-OFF time, power of 30 W) (Sonicator 4000, Misonix, with 419 Microtip probe) and DEPMPO spin trap 2% (v/v) was added to a nanotube dispersion with the final concentration of nanotubes being 1000 μg/mL. The mixture was deposited on polyethylene terephthalate (PET) slide and irradiated with UV light with the one selected wavelength within the wavelength range of 300 to 490 nm for 5 min (light current density of 0.5 W/m^2^, dose of 15 mJ). The diode was 1–2 mm away from the surface of the sample. After irradiation the samples were drawn into the capillary, which was put in the quartz sample tube for further EPR measurements.

### Cell counting using the Trypan blue

Experiment was performed in the same way as the one for cell viability test, using 1000 μg/mL of TiO_2_-NTs in the final dispersion. During the last day of the experiment, the MTS reagent application was replaced by the trypsinization of the cells (PPA Laboratories GmbH) that were then counted manually using Trypan blue (Gibco, Life tehnologies) to distinguish between live and death cells. Experiment was repeated two times to derive the mean values as well as the standard errors.

### Clonogenic test

During the last day of the treatment the cells were firstly trypsinized. Then, 100, 200 or 300 MCF-7 cells were seeded in pools (six-well plates, TTP Techno Plastic Products AG) in 1.8 mL of the cell medium with 10% of the FBS and allowed to grow for 14 days (without stressors). Finally, the colonies were counted after staining with Crystal violet (Sigma Aldrich, St. Louis). Only colonies with more than 50 cells were scored as survivors. Survival factor (SF) was calculated as the percentage of the plating efficiency (PE) of the treated cells over the plating efficiency of the untreated cells. Each experiment was repeated in 36 different wells.

### Sedimentation study

The TiO_2_-NTs dispersion with a concentration of 1000 μg/mL was sonicated to disperse the nanotubes in a medium with or without the FBS. A dispersion was then measured on an UV-VIS absorption spectrometer (Perkin-Elmer Lambda 17, program Lambda 35) at 400 nm for up to 24 h. An UV Cuvette semi-micro (Brand, BrandTech Scientific) with 10 mm light path was used. The data were background- and offset-corrected and measured at room temperature. Due to the aggregation and the accompanied sedimentation of the nanotubes the UV-VIS signal of the stably dispersed nanotubes decreases with time allowing us to interpret the absorption decrease rate as a sedimentation rate. The results (the sedimentation rates) are given as mean values of the three independent experiments together with the standard deviations. Hydrodynamic sizes of the nanotubes were measured using dynamic light scattering (DLS, ZetaSizer Nano ZS) at 173° scattering angle. Dispersion of the nanotubes at different concentrations of the FBS were centrifuged at 15000 rpm (r = 6.2 cm) for 20 min to remove the unstable aggregates. The amount of unstable aggregates was measured using PVC Packed Cell Volume Tubes (TPP, product no. 87001). The measurements of the supernatants were performed at 25°C and average hydrodynamic diameters of the nanotubes were determined with the software provided by the manufacturer of the DLS apparatus.

### Surface functionalization and fluorescence labeling of TiO_2_-NTs

**Fig 1:**
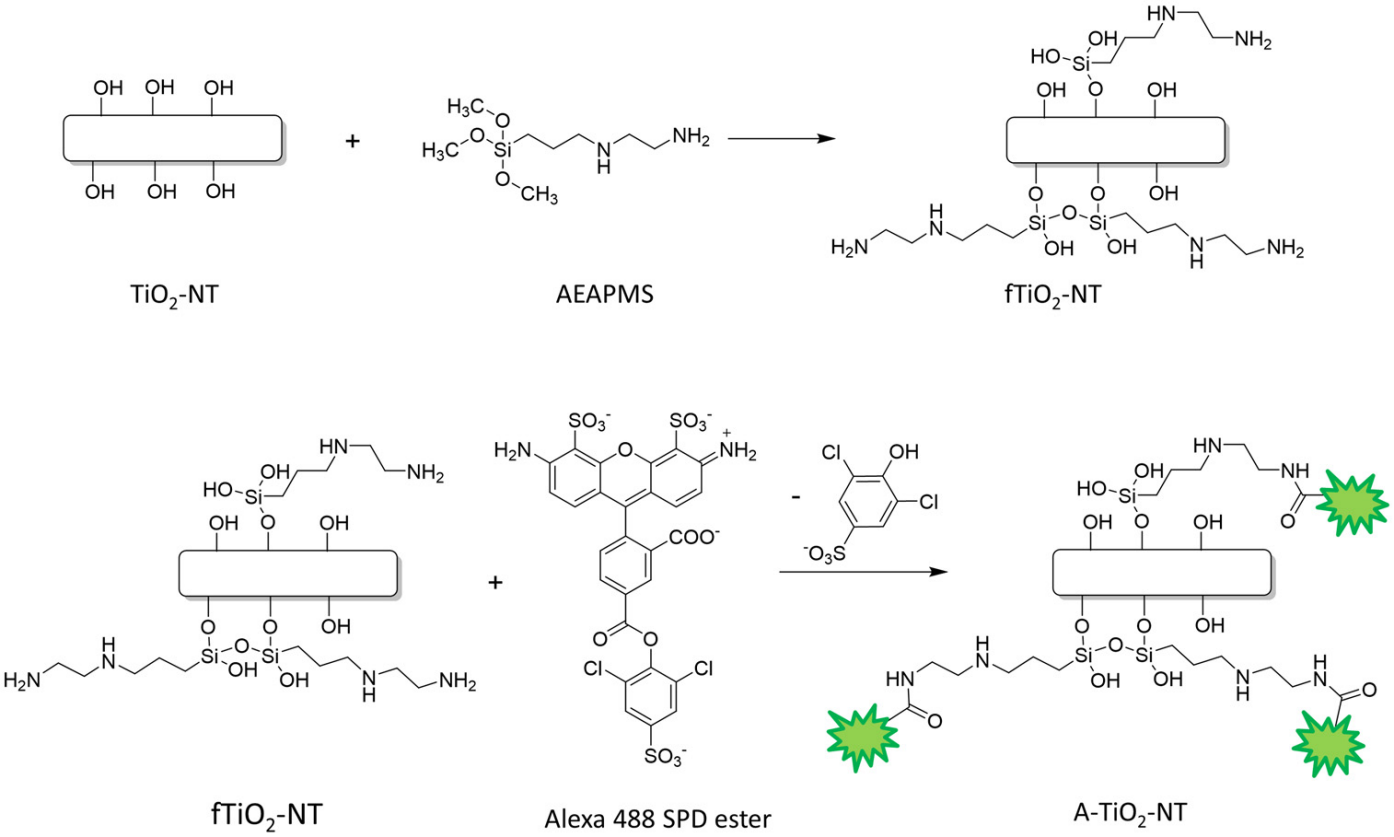
Labelling of TiO_2_-NTs with fluorophore Alexa via the two steps-reaction of the TiO_2_-NTs surface modification. Firstly, 3-(2-aminoethylamino)propyltrimethoxysilane (AEAPMS) is attached to free–OH groups of the nanotubes’ surface (fTiO_2_-NT). Secondly, the Alexa 488 SDP ester is covalently linked to the free amino groups of silane molecules (A-TiO_2_-NT).

To bind a fluorescent probe on the inorganic TiO_2_-NTs, they should be functionalized first. For this purpose, 840 μL of the silane linker, 3-(2-aminoethylamino)propyltrimethoxysilane (AEAPMS) 1.028 g/mL was dispersed in 60 mL of a dry toluene, and a droplet of AEAPMS solution was added to a dispersion of 50 mg of TiO_2_-NTs in 60 mL of a dry toluene heated at 60°C. After 16 h the sample was centrifuged and washed with 10 mL of toluene for three times. Finally, the sample was centrifuged once more, dispersed in 15 mL of ethanol and dried at 100°C in a vacuum drier at 200 mbars. An AEAPMS-functionalized TiO_2_-NT is denoted as fTiO_2_-NT (Fig 1). In the next step, the fTiO_2_-NTs were dispersed in 7 mL of the bicarbonate buffer (pH 8.4) to the final concentration of fTiO_2_-NTs of 1 mg/mL, and sonicated for 15 min in an ice-bath with the MISO XVIX Ultrasound liquid Processors with 419 Microtip probe at a power 20–30 W. The dispersion was then incubated with 20 μL of 12 mM Alexa 488 sulfo-di-chlorophenol ester in DMSO, sonicated for 2 h on an ice bath and left stirring overnight at room temperature. To remove the unbound fluorescent probe, the mixture was dialyzed for 24 h in 1 L of ethanol four times. After the fifth day ethanol was exchanged with distilled water for a day and the sample was stored at 4°C [16]. A fluorescently-labelled fTiO_2_-NT is denoted as A-TiO_2_-NT (Fig 1). In order to confirm successful functionalization and labeling of the TiO_2_-NT we used several experimental techniques as described in S5 Information. Characterization of surface modified TiO_2_-NTs (fTiO_2_-NTs) and fluorescently labeled TiO_2_-NTs (A-TiO_2_-NTs).

### Binding of the bovine serum proteins to TiO_2_-NTs

For this purpose the fluorescently-labelled nanotubes A-TiO_2_-NTs dispersed in water with the final concentration of 1000 μg/mL were sonicated for 15 min on an ultrasonic bath (Bransonic ultrasonic cleaner, Branson 2510EMT). 100 μL of this dispersion was pipetted in one well of 96-well black plate and titrated with the fetal bovine serum (FBS) in steps of 2 μL up to the final volume of 150 μL. Fluorescence emission spectra were measured during each step at the room temperature on a microplate reader Infinite M1000. Fluorescence was excited at 250 nm and emission spectra were recorded from 280 to 660 nm. The analogous experiment was performed with the free fluorophore Alexa 488 SDP dye, diluted in water, with the concentration of 0.6 μM, which is comparable to the Alexa bound within the A-TiO_2_-NTs sample. All the experiments were independently repeated twice.

### Measurements of the reactive oxygen species (ROS) by the spin trapping electron paramagnetic resonance (EPR) method

For the detection of the short lived radicals produced by UV irradiation of the TiO_2_-NTs electron paramagnetic resonance spectroscopy (EPR) was used together with spin traps. The role of a spin trap is to capture the short lived radicals and transform them into more stable radical adduct, which can be detected by EPR. EPR spectra intensity is therefore proportional to the amount of radical captured, while the spectral line-shape defined by the hyperfine splitting constants of the spin adducts identifies the type of the radical that is captured on a spin trap.

### Influence of serum proteins on the photocatalytic activity of TiO_2_-NTs

**Fig 2:**
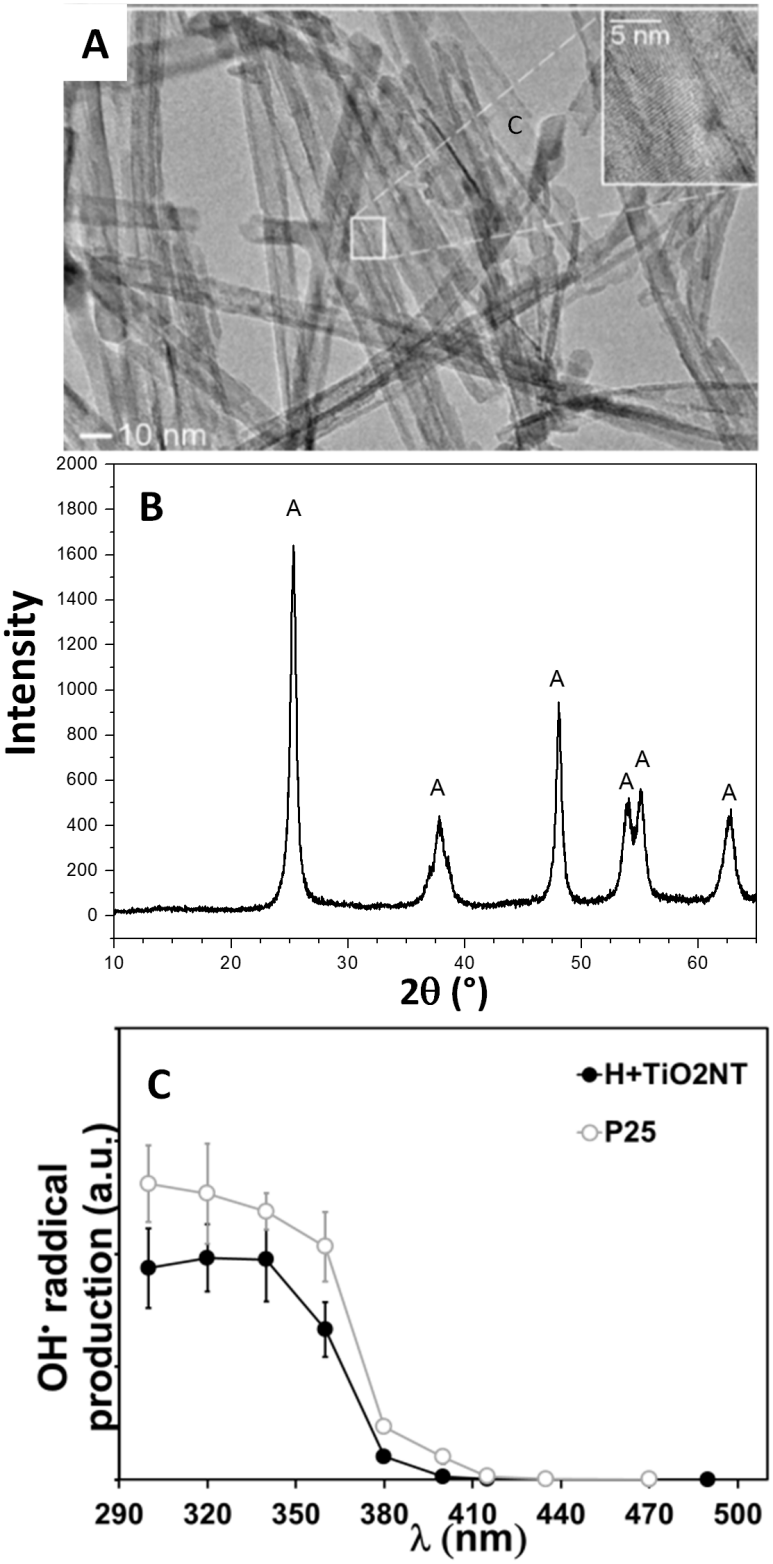
Characterization of the TiO_2_-NTs and their photo-catalytic activity. (A) TEM image of the TiO_2_-NTs. Inset in Fig 2A shows interlayer spacing of about 0.35 nm that agree well with [100] diffraction peak of anatase TiO_2_. (B) X-ray powder diffraction of TiO_2_-NTs, anatase peaks are marked with A. (C) OH• radical production of TiO_2_-NTs (closed black circles) as a function of wavelength compared to the radical production by P25 from Degussa (open circles). At 365 nm production of radicals decreases to half of their maximal activity at lower wavelengths.

For these experiment a stock dispersion of TiO_2_-NTs was diluted with DMEM cell medium without or with 10% of the FBS and 2% (v/v) of the DMPO spin trap with the final concentration of nanotubes being 1000 μg/mL. The spin-traps stock solutions were always freshly prepared. Each sample was irradiated with UV light at the wavelength of 365 nm for 5 min with the light current density of 0.5 W/m^2^ and a dose of A = 15 mJ. The wavelength was chosen on the basis of previous experiments as a compromise between efficient radical production under irradiation of TiO_2_-NTs (Fig 2C) and minimal cytotoxicity caused by UV light alone. EPR measurements were performed on the X-band EPR spectrometer Bruker ELEXYS E500 at room temperature with modulation amplitude of 0.15 mT, modulation frequency of 100 kHz, microwave power of 20 mW and thesweep width of 10 mT. The spectra were simulated with hyperfine splitting constants: A_N_ = 1.49 mT and A_H_ = 1.49 mT, typical for DMPO-OH radical adduct. From the intensity of the EPR spectra information about the amount of radical formation is obtained. To prove that DMPO-OH adducts arise from the primary generated hydroxyl radicals, which are produced by the UV irradiated TiO_2_-NTs and not due to the disintegration of DMPO, the same experiments were performed in the presence of 30% (v/v) of ethanol. The results presented in S1 Information prove that the primary radical is really the OH radical.

## Results and Discussion

### Toxic and Phototoxic effect of TiO_2_-NTs

The toxic and the phototoxic effect of the previously characterized TiO_2_-NTs [13] was investigated on the human breast adenocarcinoma cells (MCF-7). The basic morphological characterization of the TiO_2_-NTs was done by the transmission electron microscopy (TEM) (Fig 2A), which reveals the diameter and the length of the nanotubes to be around 10 nm and few hundred nanometers, respectively, being approximately the same as for sodium titanate nanotubes [14]. In addition, the X-ray powder diffraction (XRD) (Fig 2B) identifies anatase form of the TiO_2_ according to the JPCD card No. 86–1157. Photocatalytic activity of the TiO_2_-NTs was measured as a function of UV wavelength (UV LED lamp with 1.6 W/m^2^, wavelength of 365 nm) by a spin trapping electron paramagnetic resonance (EPR) technique using DMPO spin traps. Production of radicals by UV irradiation of TiO_2_-NTs is comparable to widely used P25 Degussa nanoparticles (Fig 2C).

**Fig 3:**
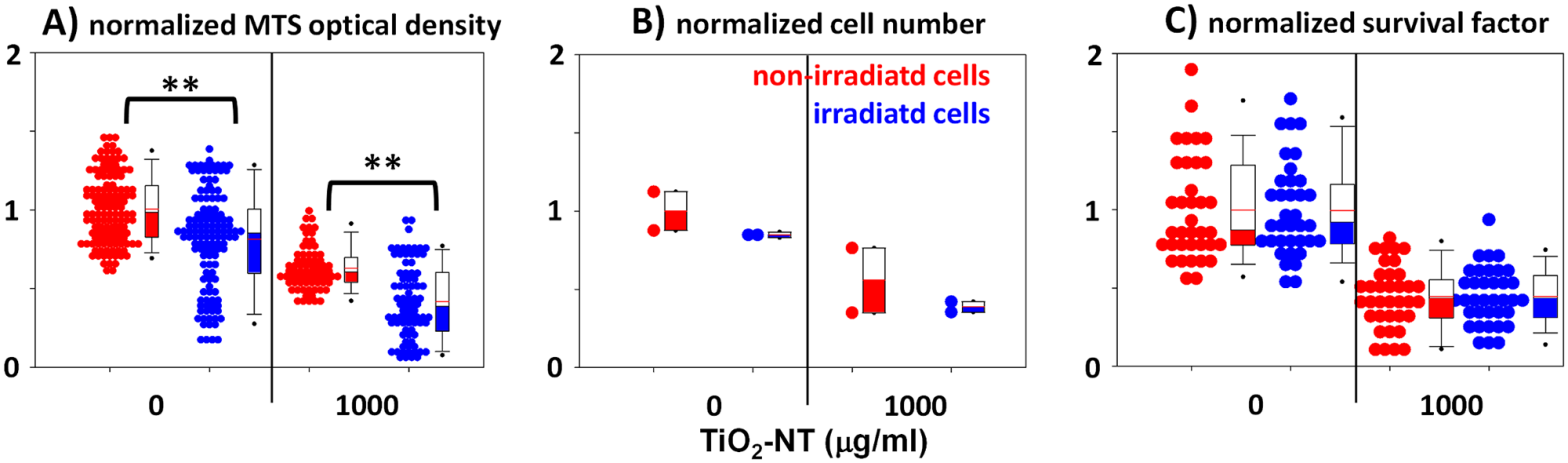
Toxicity and phototoxicity of TiO_2_-NTs. MCF-7 cells were grown during the first day, exposed to 1000 μg/mL of nanotubes during the second day and incubated in dark or under UV irradiation (1.6 W/m^2^, wavelength 356 nm, every 3 h for 15 min in a period of 24 h) during the third day. Control experiment were performed without the appropriate stressor (nanotubes during the second day or UV irradiation during the third day). On the fourth day: (A) optical density of samples was measured by MTS assay, (B) cells were trypsinized and counted manually using Trypan Blue, (C) the same number of MCF-7 cell were seeded in pools to grow for additional 14 days and colonies were counted. Survival factor (SF) was calculated as percentage of plating efficiency (PE) of the treated cells over PE of the untreated cells. Since normality test (Shapiro-Wilk) failed, we used Mann-Whitney U statistics instead. ** denotes the significant difference in the median values at P = <0.001.

The toxicity and the photo-toxicity of the TiO_2_-NTs was evaluated by MTS assay [17], giving the information about the combined effect of MCF-7 cell number and metabolic activity of MCF-7 cells (Fig 3). Although the MTS assay is sensitive and fast, it cannot distinguish between a sample with many cells of low metabolic activity and a sample with few cells of high metabolic activity. To resolve the MTS results the cell counting and the clonogenic assay have been applied. Live cells were counted using Trypane blue dye (Fig 3B) while the ability of the MCF-7 cells to divide were determined via survival factor defined as normalized plating efficiency (Fig 3C, normalized survival factor). Since the survival factor is clearly independent of UV irradiation, regardless the presence of nanoparticles, we can conclude that the absorbance measured by MTS in our case is a relevant measure of the number of cells (cell viability) and that a potential photo-toxic effect of the TiO_2_-NTs at concentration 1000 μg/mL is not related to the cell’s ability to divide.

**Fig 4:**
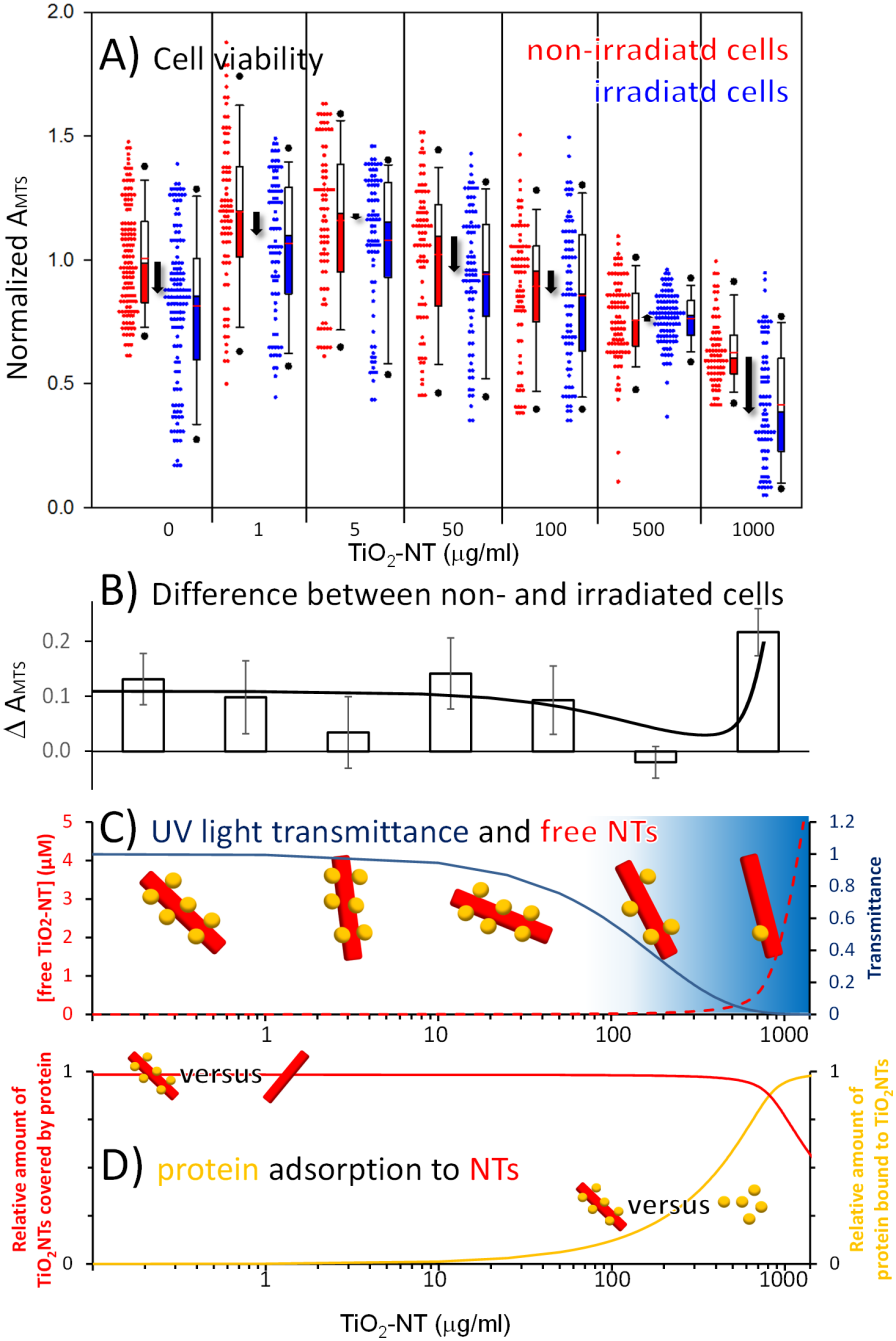
Cell viability versus concentration of TiO_2_-NTs in absence or presence of UV radiation. Absorbance of MTS was measured as described in Materials and methods section. (A) Each non-irradiated data set (red symbols) is compared to a sample irradiated with UV light (blue symbols) at given concentration of TiO_2_-NTs. All Measurements are presented in a boxplot representing the distribution of the measured cell viability and described in Materials and methods. Differences in the cell viability between irradiated and non-irradiated cells are shown with the black arrows at each concentration of TiO_2_-NTs. The differences in the median values between the two groups at 0 and 1000 μg/mL and the two groups at 1 and 50 μg/mL of nanotubes are considered to be significant at P = <0.001, and P = <0.05, respectively. (B) Differences of median values of MTS absorbance between irradiated and non-irradiated cells, shown with the black arrows within the frame A, are plotted with the open bars. Solid black line represents the prediction of the difference in cell viability, based on the effect of the UV irradiation and phototoxic effect due to irradiated nanotubes not covered by serum proteins. Both contributions are shown separately in the frame C. (C) Solid dark blue line shows the transmittance of UV light versus the concentration of the nanotubes obtained experimentally (S2 Information. Optical properties of TiO_2_-NTs dispersion.), while dashed red line represents the concentration of the free nanotubes calculated as described in the S3 Information. Model of albumin binding to TiO2-NTs and best fit parameter values. (D) The relative amount of TiO_2_-NTs covered by the serum proteins (red line) and the relative amount of serum proteins bound to TiO_2_-NTs (yellow line) are shown as predicted by the model described in the S3 Information. Model of albumin binding to TiO2-NTs and best fit parameter values.

With the reduction of the MTS signal being a consequence of the reduced cell number (viability) and not weakened metabolic activity of survived cells, we can therefore use the MTS test to determine the viability of MCF-7 cells, i.e. toxicity also at lower concentrations of the nanotubes. The test resolves the significant decrease of the cell viability only at concentrations of TiO_2_-NTs higher than 100 μg/mL (red boxes in Fig 4A). At the lowest concentration of the nanotubes (1 and 5 μg/mL) even an increase in cell viability is observed, which can be understood in terms of hormesis, where low concentration of a substance stimulates cell growth, while an inhibitory effect appears at higher concentration [18]. The phototoxicity of the TiO_2_-NTs was determined via periodic irradiation of the cells with low intensity UV light for 24 h (wavelength of 365 nm, 15 min every 3 h during 24 h, light density of 1.6 W/m^2^). The choice of 365 nm as the wavelength of UV light was selected in order to achieve satisfactory radical production under illumination of TiO_2_-NTs (Fig 2C) at the minimal cytotoxic effect caused by UV light alone. UV light alone shows a reduced cell viability at the maximal level of around 15%, the same as the UV-irradiated TiO_2_-NTs at low-concentrations (Fig 4B). At higher concentrations, the nanotubes absorb the UV light so efficiently, that photo-toxicity caused by the radical production is completely eliminated at the TiO_2_-NTs concentration of 500 μg/mL. (Fig 4C, blue line). If this was the only relevant phenomenon, one would expect the photo-toxicity to diminish at higher concentration as well. On the contrary, at the concentration of 1000 μg/mL photo-toxicity increase dramatically indicating that there is another concentration-dependent phenomenon that hinders the apparent radical production at lower concentrations. We associate the decrease of the detected radicals to the only component of the serum that can scavenge the radicals—the serum proteins, which can bind to the surfaces of the nanotubes and form a protein corona [19,20].

If photo-generation of radicals is hindered by a protein corona, then the difference in cell viability, shown in Fig 4B, will be a sum of at least two contributions. The first one is the reduction of the cell viability due to UV light alone, being proportional to transmittance of cell medium containing the nanotubes (blue line in Fig 4C). The second contribution, on the other hand, originates in the photo-induced radical generation. Undoubtedly, the amount of radicals attacking the cells is proportional to the amount of light available, i.e. transmittance of the medium with the nanotubes. In addition, the amount of radicals is further decreased via radical trapping by the serum protein coating the nanotube surface. The efficient radical release is therefore proportional to the surface not covered by the serum proteins, i.e. the concentration of the free nanotubes (red dashed line in Fig 4C). With fixed amount of the serum proteins (10% FBS, 57 μM), radical production is hindered up to the nanotube concentration (between 500 and 1000 μg/mL) at which there is not enough serum proteins to cover the TiO_2_-NTs surface and scavenge the majority of the radicals anymore (Fig 4D, red line). With respect to the serum proteins, most of the proteins are free at low concentration of TiO_2_-NTs, since there is not enough TiO_2_-NTs surface present, to which proteins could bind to (Fig 4D, yellow line). However, almost all the serum proteins bind to the TiO_2_-NTs surface when the concentration of the TiO_2_-NTs approaches about 500 μg/mL, leaving a free TiO_2_-NTs surface at even higher concentrations.

### Interaction of TiO_2_-NTs with serum proteins

Possible interactions between the TiO_2_-NTs and proteins of the fetal bovine serum (FBS) that should result in a formation of the protein corona around the nanotubes [21–25] have been studied through the effects on the stability of the dispersion, the fluorescence quenching and the radical scavenging.

**Fig 5:**
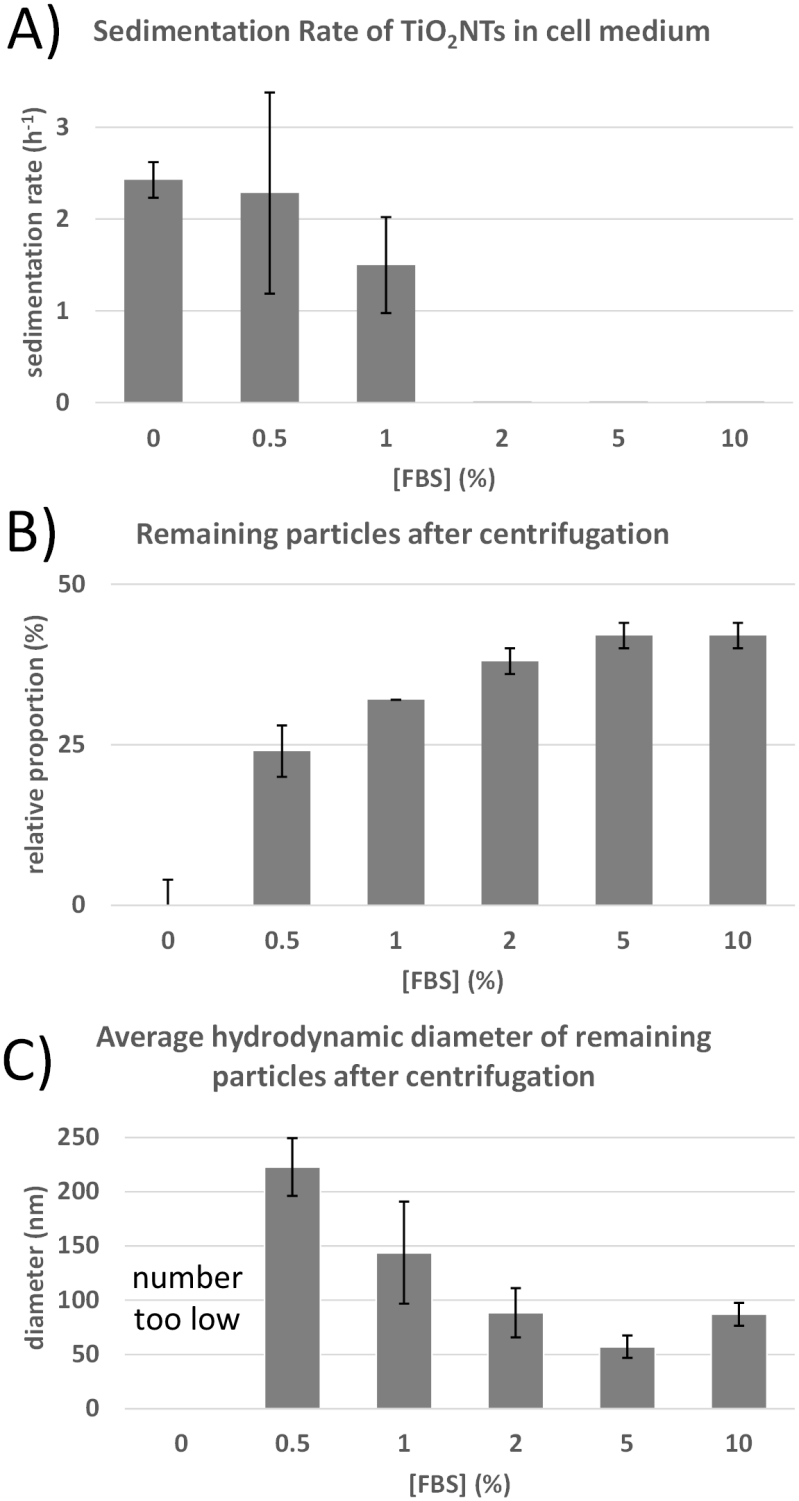
Stability of the TiO_2_-NTs in cell medium at different concentrations of FBS. (A) Rate of sedimentation measured through the absorbance at 400 nm on a UV-VIS spectrometer up to one day. (B) Relative proportion of the remaining TiO_2_-NTs in dispersion after centrifugation at 15000 rpm (r = 6.2 cm) for 20 min. (C) Average hydrodynamic diameter of the remaining small aggregates and coated single nanotubes in dispersion of the TiO_2_-NTs after centrifugation.

The TiO_2_-NTs dispersion is known to be unstable. With the precipitation of the large aggregates of the nanotubes, the concentration of the individual nanotubes and the small aggregates should decrease rapidly. On the other hand, a dispersion of the serum proteins is much more stable due to the negative charge. The latter introduces electrostatic repulsion that can prevail over the Van Der Waals attraction between the proteins. Binding of the serum proteins to the nanotubes is therefore expected to influence the stability of the nanotube dispersion. To investigate the latter the absorbance has been measured in the cell medium containing 1000 μg/mL of the nanotubes and varying concentration of the serum proteins (up to 10% FBS, which is equal to 57 μM of albumin). In absence of FBS the absorption of light decreases below 50% in less than half an hour, indicating an intensive precipitation of the nanotube (S4 Information. Sedimentation of TiO2-NTs dispersion.). While increasing the FBS concentration, the sedimentation rate, at which light absorption decreases to a half, decreases (Fig 5A). Concentrations of FBS of 2% and more completely prevent the precipitation during the first 24 h, supporting the idea that proteins can stabilize nanotubes in the dispersion by preventing aggregation, being in agreement also with the previous studies [26]. When the dispersion of the nanotubes is centrifuged the amount of the nanotubes remaining stably dispersed increases with an increasing amount of the FBS (Fig 5B). In accordance with this result, the average hydrodynamic diameter of the nanotube aggregates being stably dispersed decreases (Fig 5C). This indicates that at least 2% FBS is needed to stabilize the dispersion of 1000 μg/mL of the nanotubes.

**Fig 6:**
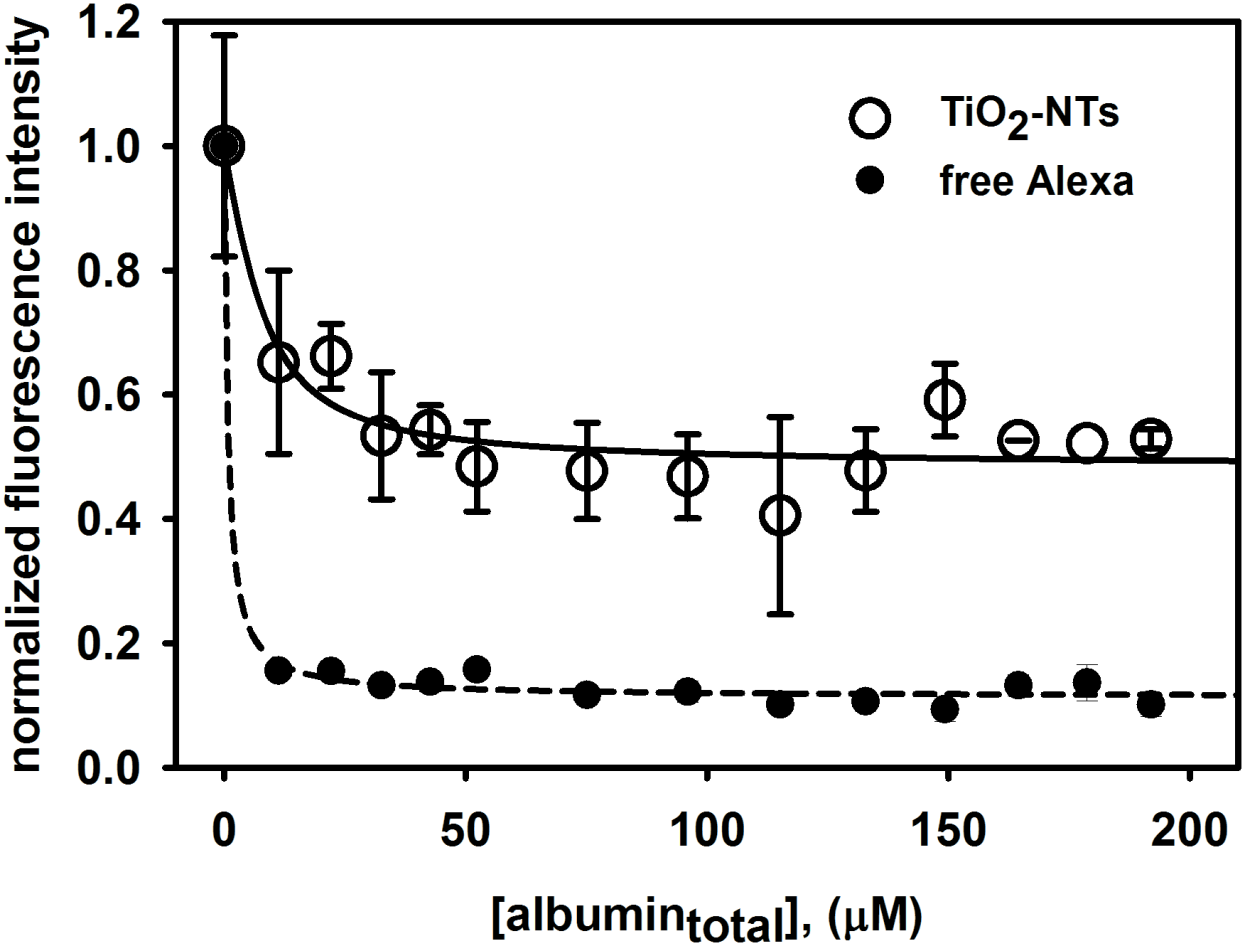
Fluorescence quenching due to binding of the bovine serum albumin to fluorescently labelled A-TiO_2_-NTs. Intensity of fluorescence emission of Alexa labeled nanotubes excited at 250 nm was measured on spectrofluorimeter. Fluorescently labeled nanotubes (A-TiO_2_-NTs) were dispersed in water (300 μg/mL) and sonicated on ultrasonic bath, then 100 μL of dispersion was titrated with FBS in 2 μL steps to final volume 150 μL. The analog experiment with 0.6 μM Alexa has been done for reference to prove that the free Alexa has a distinguishable titration curve from the bound Alexa.

The increase in the stability of the dispersion undoubtedly indicates that proteins form at least an electrical double layer around the nanotubes and affect the Zeta potential of the nanotubes. However, the stability measurements cannot prove that the proteins are actually bound to the nanotube surfaces. To characterize an average distance between the serum proteins and the nanotubes in a coated nanotube formation, we fluorescently labelled the nanotubes with Alexa fluorophore (A-TiO_2_-NTs) as described in the materials and methods section (Fig 1) and followed the quenching of the nanoparticle-bound Alexa fluorescence with proteins as described in [27]. A decrease of fluorescence emission intensity of bound Alexa was observed (Fig 6, white dots) for the concentration of 300 μg/mL of A-TiO_2_-NTs exhibiting saturation-like response. The surface of the nanotubes is fully coated by the proteins at the protein concentration of 50 μM and above. On the other hand, to get significantly uncoated nanotubes in the dispersion of 300 μg/mL the protein concentration must fall below 20 μM. At the protein concentration of 57 μM, which is used in our experiment, it is thus reasonable to observe a significant effect of the free nanoparticle surface only at the highest nanotube concentration of 1000 μg/mL (Fig 3). Since the free Alexa dye (at the concentration of 0.6 μM) is quenched much more efficiently by the serum proteins than the nanotube-bound Alexa and the two titration curves are so significantly different from each other, we can conclude that Alexa fluorophore is tightly bound on the nanotubes. Taking into account that the quenching can be detected only when the quencher forms a molecular contact with the fluorophore, this experiment confirms that the serum proteins most probably bind to the nanotubes.

We estimate that up to 50 albumin molecules (albumin nevertheless presents 74% of total protein) are needed to fully cover a single TiO_2_ nanotube, taking into account dimensions of albumin and the surface area of an average TiO_2_ nanotube estimated from the TEM images (Fig 2). Using a simple binding model, which takes into account that *N* molecules of albumin bind to a nanotube, as explained in the S3 Information. Model of albumin binding to TiO2-NTs and best fit parameter values, we estimated that 1 to 7 albumin molecules bind to one nanotube with dissociation constant in the range between 1 to 4 μM (S1 Information). With these two parameters, we estimated that at 1000 μg/mL of the nanotubes 10–30% of the surface of nanotubes should be uncovered by the serum proteins. This predicts that protein covered nanotubes should have only 10–30% of the photocatalytic activity of the nanotubes not covered by proteins. To test this estimation, we used the spin trapping EPR spectroscopy.

### Free radical formation by free or protein coated nanotubes

**Fig 7:**
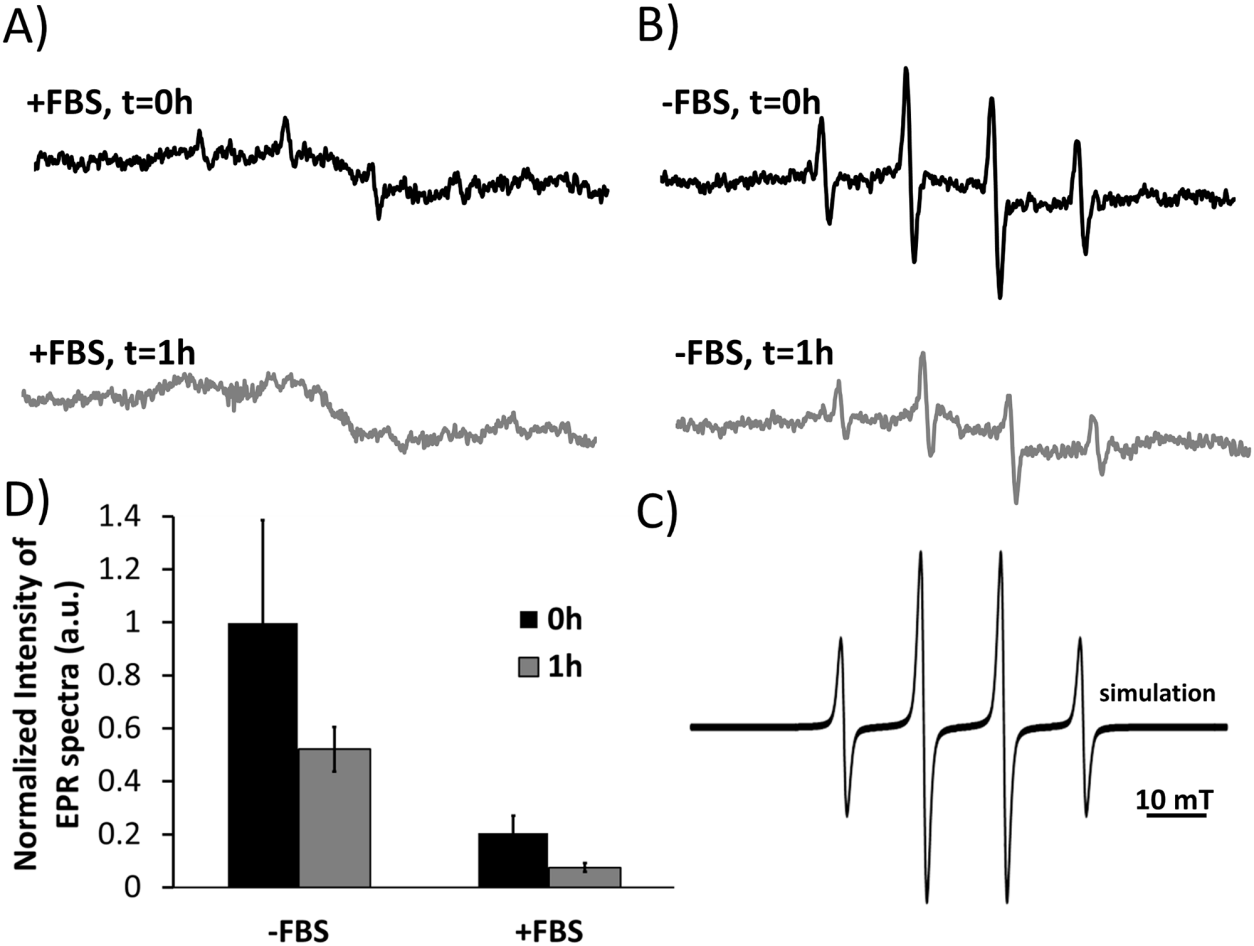
Measurements of hydroxyl radical formations by UV irradiation of the TiO_2_-NTs. Spin trap DMPO was used to detect production of the hydroxyl radicals generated by UV irradiated TiO_2_-NTs. TiO_2_-NTs were mixed with DMPO spin traps and cell medium with 10% FBS or without FBS. The sample was irradiated for 5 min with UV light (wavelength of 356 nm) or left in dark (control), followed by EPR measurements immediately after the addition of the cell medium. In parallel experiments the dispersion of the TiO_2_-NTs in the cell medium without or with the serum proteins (FBS) was put in the dark for one hour, than the spin trap DMPO was added and samples were irradiated with the UV light. (A) Representative EPR spectrum of a trapped hydroxyl radical in the presence of the FBS or (B) absence of the FBS. (C) The experimental EPR spectrum was simulated with hyperfine splitting constants: A_N_ = 1.49 G and A_H_ = 1.49 mT typical for OH radical. Simulation was done with EPRSim Wizard [29]. EPR spectrum intensity peak normalized to the experiment with highest intensity peak, of nanomaterial in the cell medium without the FBS and with the FBS.

To determine the effect of the protein corona on the amount of radicals released from the surface of the TiO_2_-NTs under influence of UV light, we used a spin trapping electron paramagnetic resonance (EPR) method. Spin trap DMPO (5,5-dimethyl-pyrroline N-oxide) was employed to trap short lived hydroxyl radicals and transform them into more stable DMPO-OH· adducts. EPR spectra of the latter have been acquired in the presence (Fig 7A, +FBS) and the absence of the serum proteins (Fig 7B, -FBS) and their intensity has been determined. In both cases, the EPR spectra characteristic for DMPO-OH· radical are obtained, as it is proved by the simulation of EPR spectra (Fig 7C). The intensity of the EPR spectra is proportional to the number of DMPO-OH· adducts generated within the given amount of time decreased by the number of the radicals captured before reaching the DMPO spin traps. Since the number of radicals detected by the DMPO trap decreases to about 20% due to the presence of the serum proteins (Fig 7D, black bars), this can only indicate that about 80% of the generated radicals have been trapped by other than the DMPO spin traps. Taking into account the very high concentration of the DMPO trap and the corresponding volume per one DMPO molecule, we can conclude that about 80% of the nanotubes’ surface is covered by the serum proteins that are less than a nanometer away from the nanotubes’ surface. Taking into account that uncoated TiO_2_-NTs precipitate much faster than the coated ones (Fig 5), the amount of spin trapped radicals is expected to decrease with time proportionally to the number of uncoated nanoparticles, which precipitate from the sensitive area of the EPR resonator. Since this is exactly what can be measured in the experiments (Fig 7D, grey bars), this fully supports the hypothesis that the bound serum proteins do mediate radical production and sedimentation of the TiO_2_-NTs.

Spin traps like DMPO are highly reactive compounds which can form nitroxides similar to DMPO-OH adducts by mechanisms other than OH radical trapping [28]. To avoid artefacts the control experiment was repeated in 30% (v/v) of ethanol (EtOH), which becomes a primary spin trap, traps the photogenerated hydroxyl radicals, and transforms them into hydroxyethyl radicals resulting in a typical hydroxyethyl-DMPO adduct (S1 Information. DMPO-OH adduct originate from primary OH radical formed after UV-irradiation). Absence of DMPO-OH spectra in this case confirms that DMPO spin traps in the original experiment really trap the photogenerated OH radicals and not some other reactive compounds.

## Conclusions

- TiO_2_-NTs have low toxicity against human breast adenocarcinoma cells (MCF-7) with LC50 above 1000 μg/mL.
- Phototoxic effect of TiO_2_-NTs was observed at the concentration of the nanotubes of 1000 μg/mL when the serum protein concentration was too low to fully cover the nanotubes’ surface.
- TiO_2_-NTs have been successfully fluorescently labeled to study nanoparticle-protein interactions.
- The serum proteins bound to the TiO_2_-NTs nanotubes stabilize the dispersion of TiO_2_-NTs and scavenge photogenerated radicals, thus preventing phototoxic effect of UV irradiated nanotubes.
- TiO_2_-NTs are promising for the photodynamic cancer therapy as an inherently not toxic material, with its phototoxicity tunable by a low intensity UV-A light and with the relative coverage of their surface by the serum proteins.

## Supporting Information

S1 Information DMPO-OH adduct originate from primary OH radical formed after UV-irradiation. (DOCX)

S2 Information Optical properties of TiO_2_-NTs dispersion. (DOCX)

S3 Information Model of albumin binding to TiO_2_-NTs and best fit parameter values. (DOCX)

S4 Information Sedimentation of TiO_2_-NTs dispersion. (DOCX)

S5 Information Characterization of surface modified TiO_2_-NTs (fTiO_2_-NTs) and fluorescently labeled TiO_2_-NTs (A-TiO_2_-NTs). (DOCX)
