# Supplementary material for: Protein Corona Prevents TiO2 Phototoxicity
Source: PLoS One. 2015 Jun 17;10(6):e0129577. doi: 10.1371/journal.pone.0129577 (PMC4470505; doi:10.1371/journal.pone.0129577)
Supplement: S1 Information — (DOCX) [file pone.0129577.s001.docx]

**S1 Supporting Information. DMPO-OH adduct originate from primary OH radical formed after UV-irradiation.**

If the production of DMPO-OH is due to the spin trapping of hydroxyl radical, the radical would react with ethanol and produce hydroxyethyl radical, which is than trapped by DMPO and results in the appearance of a new signal.

**Figure. EPR spectra with DMPO spin trap in presence of 30% of EtOH to prove production of -hydroxyethyl** radical **[1]**. 1000 μg/mL of TiO_2_-NTs in DMEM with 10% of FBS, 30% of EtOH and DMPO was irradiated with 365 nm UV LED lamp for 5 minutes. On the spectrum two radical adducts are resolved, which could be simulated with hyperfine splitting constants: domain 1: typical for OH adduct and domain 2 typical for hydroxyethyl radical adduct.

Decomposition of simulated spectrum nicely showed presence of -hydroxiethyl radical which proves that after UV irradiation of TiO_2_-NTs hydroxyl radicals are formed (Domain 2). Small fraction of DMPO-OH radical indicate that during irradiation also some decomposition of DMPO occurred.

Literature:

1. Adams GE, Wardman P. CHAPTER 2 - Free Radicals in Biology: The Pulse Radiolysis Approach. In: Pryor WA, editor. Free Radicals in Biology. Academic Press; 1977. pp. 53–95.
