# Supplementary material for: Protein Corona Prevents TiO2 Phototoxicity
Source: PLoS One. 2015 Jun 17;10(6):e0129577. doi: 10.1371/journal.pone.0129577 (PMC4470505; doi:10.1371/journal.pone.0129577)
Supplement: S2 Information — (DOCX) [file pone.0129577.s002.docx]

**S2 Supporting Information. Optical properties of TiO_2_-NTs dispersion.**

**Figure. Absorbance of TiO_2_ nanotubes dispersion versus concentration of the nanotubes.** TiO_2_-NTs powder was dispersed in KOH, sonicated and then diluted in distillated water to final concentrations between 200 and 1000 μg/ml. Absorbance was measured at 400 nm on a UV-VIS spectrometer .Results are averages of two independent experiments.
