# Supplementary material for: Protein Corona Prevents TiO2 Phototoxicity
Source: PLoS One. 2015 Jun 17;10(6):e0129577. doi: 10.1371/journal.pone.0129577 (PMC4470505; doi:10.1371/journal.pone.0129577)
Supplement: S3 Information — (DOCX) [file pone.0129577.s003.docx]

**S3 Supporting Information. Model of albumin binding to TiO_2_-NTs and best fit parameter values.**

**Binding of N albumin molecules to a nanotube**

The simplest model describing albumin binding to a TiO_2_ nanotube (TiO_2_-NT) is based on the hypothesis that N albumins bind to a nanotube. The observed fluorescence intensity (FI) of fluorescently labelled TiO_2_ nanotubes is then the sum of fluorescence intensities of only two species: $FI=[\mathrm{TiO}_{2}\mathrm{NT}_{free}]\cdot F_{TiO_{2}NT}+[alb\mathrm{TiO}_{2}\mathrm{NT}]\cdot F_{albTiO_{2}NT}$, A1

where $[\mathrm{TiO}_{2}\mathrm{NT}]$ and $[alb\mathrm{TiO}_{2}\mathrm{NT}]$represent the concentration of free and TiO2 nanotubes with N albumins bound, respectively. $F_{TiO_{2}NT_{free}}$and $F_{albTiO_{2}NT}$ represent the intrinsic fluorescence of the two TiO_2_ nanotube species. It is useful to normalize the fluorescence intensity to the fluorescence signal of free nanotubes:

$FI_{norm}=\frac{[\mathrm{TiO}_{2}\mathrm{NT}_{free}]\cdot F_{TiO_{2}NT_{free}}}{[\mathrm{TiO}_{2}\mathrm{NT}_{total}]\cdot F_{TiO_{2}NT_{total}}}+\frac{[alb\mathrm{TiO}_{2}\mathrm{NT}]\cdot F_{albTiO_{2}NT}}{[\mathrm{TiO}_{2}\mathrm{NT}_{total}]\cdot F_{TiO_{2}NT_{total}}}$, A2

The normalized fluorescence intensity *FI_norm_* depends on relative concentration of the two species and their relative fluorescence intensities with respect to the concentration of the total concentration of TiO_2_ nanotubes and their relative fluorescence intensity:

$FI_{norm}=\frac{[\mathrm{TiO}_{2}\mathrm{NT}_{free}]}{[\mathrm{TiO}_{2}\mathrm{NT}_{total}]}\cdot f_{TiO_{2}NT_{free}}+\frac{[alb\mathrm{TiO}_{2}\mathrm{NT}]}{[\mathrm{TiO}_{2}\mathrm{NT}_{total}]}\cdot f_{albTiO_{2}NT}$, A2

We know the total concentration of the nanotubes in the experiment and their relative fluorescence intensity. The unknown parameters are relative concentrations of the two species and the relative fluorescence intensity of albumin coated nanotubes, so we treat it as an adjustable parameter in our calculations.

To obtain the concentrations of the two species we consider the process of binding of albumin to a nanotube as a reaction involving the two species:
 , A2

where $TiO_{2}NT_{free}$is the concentration of free nanotubes, $alb_{free}$ is the concentration of free albumin, and $albTiO_{2}NT$ is the concentration of nanotubes with bound albumin molecules. We assume that these equilibria are established rapidly and that all protein species are in equilibrium during our measurements. Thus, the dissociation constant of the process is:

$K_{d}=\frac{[TiO_{2}NT_{free}]\cdot[alb_{free}]}{[albTiO_{2}NT]}$ A3

Since the total concentrations of $[TiO_{2}NT_{total}]$ and $[alb_{total}]$ are conserved, we can write two conservation equations:
 A5

One can then easily express concentration of nanotubes with bound albumin:

$[\mathrm{alb}TiO_{2}NT]=\frac{1}{2N}\left([\mathrm{alb}_{total}]+\mathrm{N}\cdot[TiO_{2}NT_{total}]+K_{d}-\sqrt{\left([\mathrm{alb}_{total}]+\mathrm{N}\cdot[TiO_{2}NT_{total}]+K_{d}\right)^{2}-4N[\mathrm{alb}_{total}][TiO_{2}NT_{total}]}\right)$. A6

With the concentrations of all species known, Eq. A1 provides fluorescence intensity that can be compared to experimental fluorescence intensity to obtain the number of albumin molecules bound to a nanotube *N*, and the dissociation constant of albumin binding $K_{d}$by minimizing the square deviation of predicted fluorescence intensity FI from the experimental FI_exp_. Best fit parameter values are shown in table below. The fit for this model is given in Figure 4C (red dashed line) of the manuscript.

**Table: best fit parameters.**

| **K_d_ (μM)** | **N** | $f_{albTiO_{2}NT}$ |
| --- | --- | --- |
| **4±5** | **2±5** | **0.52±0.07** |

If we plot the concentration of different species calculated with the best fit parameters versus the concentration of the nanotubes, we can see that the amount of the free nanotubes increases from 0.4 to 1 µM when total nanotube concentration increases from 5 to 10 µM (which corresponds to 500 to 1000 μg/mL) (Figure 1A, below). Keeping the fitting parameters within the range of errors: Kd=1 µM , and N=7, the model predicts that the amount of the free nanotubes can increase for ten times, from 0.2 to 2.3 µM, when total nanotube concentration increases from 5 to 10 µM (500 to 1000 μg/mL).

| A) | 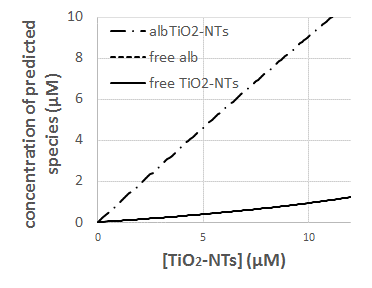 |
| --- | --- |
| B) | 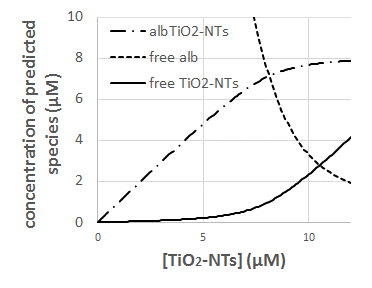 |

***Figure. Concentrations of different TiO_2_ nanotube species as predicted by the albumin to nanotube binding model.*** *The concentration of the nanotubes coated with albumin (albTiO_2_-NTs, dash-dotted line), the free nanotubes without bound albumin (free TiO_2_-NTs, solid line), and free albumin (free alb, dashed line) versus the total concentration of the nanotubes. The concentrations were determined according to the binding model, which takes into account that N molecules of albumin can bind to a nanotube (as described above). A) Predicted concentrations with parameters obtained from the best fit to the data shown in Figure 6 in the manuscript: N=2, Kd=4 µM, and B) Predicted concentrations with parameters in the range of error of the best fit: N=7, Kd=1 µM.*
