# Supplementary material for: Protein Corona Prevents TiO2 Phototoxicity
Source: PLoS One. 2015 Jun 17;10(6):e0129577. doi: 10.1371/journal.pone.0129577 (PMC4470505; doi:10.1371/journal.pone.0129577)
Supplement: S4 Information — (DOCX) [file pone.0129577.s004.docx]

**S4 Supporting Information. Sedimentation of TiO_2_-NTs dispersion.**

The TiO_2_-NTs dispersion with a concentration of 1000 μg/mL was sonicated to disperse the nanotubes in a medium with different concentration of serum proteins (FBS) or without the FBS. The TiO_2_-NTs dispersion was then measured on an UV-VIS absorption spectrometer (Perkin-Elmer Lambda 17, program Lambda 35) at 400 nm for up to 24 h. Due to the aggregation and the accompanied sedimentation of the nanotubes the UV-VIS signal of the stably dispersed nanotubes decreases with time allowing us to interpret the absorption decrease (to one half of the original intensity) as a sedimentation rate. The sedimentation rates are shown in the manuscript.

***Figure. Stability of TiO_2_-NTs dispersion in presence of serum proteins as a function of time.*** *The TiO_2_-NTs powder was dispersed in KOH, sonicated and then diluted in cell medium at different concentrations of FBS to final concentrations of 1000 μg/mL. Absorbance was measured at 400 nm on a UV-VIS spectrometer continuously for one day. The protein concentrations are shown in figure legend. Representative experiment is shown for each protein concentration.*
