# Supplementary material for: Protein Corona Prevents TiO2 Phototoxicity
Source: PLoS One. 2015 Jun 17;10(6):e0129577. doi: 10.1371/journal.pone.0129577 (PMC4470505; doi:10.1371/journal.pone.0129577)
Supplement: S5 Information — (DOCX) [file pone.0129577.s005.docx]

**S5 Supporting Information.** **Characterization of surface modified TiO_2_-NTs (fTiO_2_-NTs) and fluorescently labeled TiO_2_-NTs (A-TiO_2_-NTs).**

To confirm successful functionalization of TiO_2_-NTs infrared spectra of TiO_2_-NT and fTiO_2_-NT were recorded on a PerkinElmer spectrometer (Spectrum 400) using a KBr and a DRIFT module and a KBR module. Spectrum of 3-(2-aminoethylamino)propyltrimethoxysilane (AEAPMS) was measured using a a KBr pellet on which a drop of silan was deposited. Net charge organization of nanoparticles in water suspension was obtained by measuring -potential. Zeta-potential was measured as a function of pH of all three samples (TiO_2_-NTs, fTiO_2_-NTs and A-TiO_2_-NTs) by electro-kinetic measurements (Brookhaven Instruments Corporation, ZetaPALS). Concentration of labelled TiO_2_-NT (A-TiO_2_-NT) was estimated by an UV-VIS spectrometer (Perkin-Elmer Lambda 17). Successful labelling of TiO_2_-NT was examined by fluorescence intensity and fluorescence spectral characteristics measurements of A-TiO_2_-NT in comparison to Alexa free day. Both samples were prepared in water and were measured by a microplate reader Infinite M1000 (Tecan, Männedorf, Switzerland). The samples were pipet to 96-well black plate (BRANDplates cellGrade 96-Well Microplates, BrandTech Scientific, USA) Fluorescence of both samples was excited at 488 nm and emission light was recorded from 500 to 600 nm.

Figure 1 below shows FTIR spectra of TiO_2_-NTs (black line), functionalized nanotubes (fTiO_2_-NTs) (red line) and silane linker 3-(2-aminoethylamino) propyltrimethoxysilane (AEAPMS), used for surface functionalization (green line). Comparing spectra of TiO_2_-NTs and fTiO_2_-NTs (black with red) one can see that the small peak at 3600 cm^-1^, which corresponds to vibrations of hydroxyl groups on the surface of nanotubes [1]. In the spectrum of fTiO_2_-NTs this peak is missing, which proves efficient surface modification of TiO_2_-NTs with silane molecules. Same spectrum has peak at 2850 cm^-1^ which corresponds to C-H vibration of alkanes, peak at 1650 cm^-1^ indicate presence of amino group (NH_2_) and small, weak peak at 1260 cm^-1^ corresponds to C-N stretching.

Green line present spectrum of 3-(2-aminoethylamino)propyltrimethoxysilane (AEAPMS), used for nanotubes functionalization (green line).

**Figure 1. FTIR spectra of the TiO_2_ samples and AEAPMS.** Black line presents the spectrum of TiO_2_-NTs (the strong bend below 700 cm^-1^ corresponds to Ti-O and Ti-O-Ti bonding of titanium. The large hump between 3700 and 2600 cm^-1^ with a peak at 3250 cm^-1^ and the small peak at 1640 cm^-1^ are characteristic to stretching vibration of absorbed water. Small peak at 3600 cm^-1^ corresponds to vibrations of hydroxyl groups on the surface of the nanotubes [1] .

HAADF-STEM characterization technique combined with EEL spectroscopy was further employed to prove the surface functionalization of TiO_2_-NTs (Figure 2). Analysis of a cluster of fTiO_2_-NTs reveals presence of Ti, C and N elements in a sample. Carbon profile is well aligned with sample profile which indicates that detected carbon comes from AEAPMS and is not an artefact of a sample contamination. Nitrogen detection is very low but again is well aligned with carbon profile, which confirms successful TiO_2_-NTs surface functionalization.

**Figure 2. HAADF-STEM image of TiO_2_-NTs clusters, functionalized with 3-(2-aminoethyl amino)propyltrimethoxysilane (AEAPMS):** (A). EELS analysis of present elements (Ti L, C K and N K) in fTiO_2_-NTs sample obtained along red arrow in figure A is shown (B).

The fluorescence emission spectrum of fluorescently labelled A-TiO_2_-NTs and of free Alexa dye are presented in Figure 3 blue lambda shift of Alexa dye confirms successful labelling of TiO_2_-NTs.


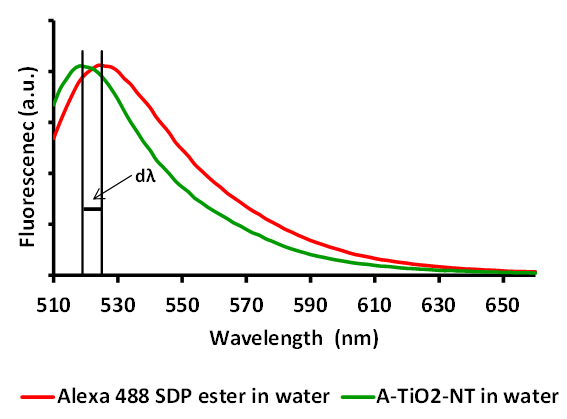


**Figure 3. Blue lambda shift (dλ) of Alexa dye, a consequence of successful labelling TiO_2_-NTs surface.** Fluorescence emission spectrum of fluorescently labelled A-TiO_2_-NTs (green) compared to emission spectrum of free Alexa dye (red) in water.

Net charge organization of nanoparticles in water suspension was obtained by measuring -potential. Zeta-potential was measured as a function of pH of all three samples (TiO_2_-NTs, fTiO_2_-NTs and A-TiO_2_-NTs) by electro-kinetic measurements (Brookhaven Instruments Corporation, ZetaPALS) (Figure 4). Result shows how material surface was changed during different steps of TiO_2_-NTs labelling. Linked nanotubes with silane linker containing amino groups on a surface resulted in positive zeta potential in pH rage till pH 9 (red dots in Figure 4). After covalent binding of Alexa 488 SDP ester to free amino group, isoelectric point of A-TiO_2_-NTs shifted to lower pH (Figure 4; green dots). After attaching Alexa molecule to amino group still poses two SO_3_^-^ groups. Comparing zeta-potential of original TiO_2_-NTs with fluorescently labelled A-TiO_2_-NTs as a function of pH (Figure 4; black and green dots, respectively) relatively good matching was confirmed. This indicates that surface charge of labelled TiO_2_-NTs is comparable to original TiO_2_-NTs.

**Figure 4. Zeta potential of original TiO_2_ nanotubes (black closed circles, TiO_2_-NTs), functionalized TiO_2_ nanotubes (red closed circles, fTiO_2_-NTs) and fluorescently labelled TiO_2_ nanotubes (green closed circles, A-TiO_2_-NTs).**

Literature:

1. Chen Q, Yakovlev NL. Adsorption and interaction of organosilanes on TiO2 nanoparticles. Appl Surf Sci. 2010;257: 1395–1400. doi:10.1016/j.apsusc.2010.08.036
